# Roux-en-Y Gastric Bypass Surgery Induces Distinct but Frequently Transient Effects on Acylcarnitine, Bile Acid and Phospholipid Levels

**DOI:** 10.3390/metabo8040083

**Published:** 2018-11-23

**Authors:** Jarlei Fiamoncini, Carina Fernandes Barbosa, José Rubens Arnoni Junior, José Celestino Araújo Junior, Cinthia Taglieri, Tiago Szego, Barbara Gelhaus, Heraldo Possolo de Souza, Hannelore Daniel, Thais Martins de Lima

**Affiliations:** 1 Department of Food Science and Experimental Nutrition, School of Pharmaceutical Sciences, University of São Paulo, 05508-060 São Paulo, Brazil; jarlei@usp.br; 2 Nutrition and Food Sciences, Technische Universität München, 85354 Freising-Weihenstephan, Germany; b.gelhaus@tum.de (B.G.); hannelore.daniel@tum.de (H.D.); 3 Clinica IMEC, Sao Paulo, 04260-020 São Paulo, Brazil; carina.fernandes@clinicaimec.com.br (C.F.B.); arnonijr@uol.com.br (J.R.A.J.); juniorcelestino@uol.com.br (J.C.A.J.); citaglieri@hotmail.com (C.T.); 4 Instituto CIGO, 05508-060 São Paulo, Brazil; ttsbrasil@gmail.com; 5 Laboratório de Emergências Clínicas (LIM 51), Hospital das Clinicas HCFMUSP, Faculdade de Medicina, Universidade de Sao Paulo, 05508-060 São Paulo, Brazil; heraldo.possolo@fm.usp.br

**Keywords:** obesity, bariatric surgery, acylcarnitine, bile acids, phospholipids, metabolomics

## Abstract

Roux-en-Y gastric bypass (RYGB) is an effective method to achieve sustained weight loss, but the mechanisms responsible for RYGB effects have not yet been fully characterized. In this study, we profiled the concentrations of 143 lipid metabolites in dry blood spots (DBS) of RYGB patients. DBS from obese patients (BMI range 35–44 kg/m^2^) were collected 7 days before, 15 and 90 days after the surgery. LC-MS/MS was used to quantify acylcarnitines, phosphatidylcholines, sphingomyelins and bile acids. RYGB caused a rapid increase in acylcarnitine levels that proved to be only transient, contrasting with the sustained decrease in phosphatidylcholines and increase of sphingomyelins and bile acids. A PLS-DA analysis revealed a 3-component model (R^2^ = 0.9, Q^2^ = 0.74) with key metabolites responsible for the overall metabolite differences. These included the BCAA-derived acylcarnitines and sphingomyelins with 16 and 18 carbons. We found important correlations between the levels of BCAA-derived acylcarnitines and specific sphingomyelins with plasma cholesterol and triacylglycerol concentrations. Along with the marked weight loss and clinical improvements, RYGB induced specific alterations in plasma acylcarnitines, bile acid and phospholipid levels. This calls for more studies on RYGB effects aiming to elucidate the metabolic adaptations that follow this procedure.

## 1. Introduction

Obesity represents a major health challenge, whose main consequence is the increase in the prevalence of metabolic diseases such as metabolic syndrome, type 2 diabetes, cardiovascular diseases, hepatic steatosis, and different types of cancer [1]. Obesity and the comorbidities pose a huge burden for health systems demanding efficient treatment options [2].

Bariatric surgery is the most efficient therapy against obesity, with four dominant surgical procedures: biliopancreatic diversion, roux-en-Y gastric bypass (RYGB), adjustable gastric banding and sleeve gastrectomy, ranging from malabsorptive to completely restrictive [3]. The procedures induce effects beyond body weight reduction, including resolution or improvement of comorbidities such as diabetes, hypertension, hyperlipidemia and sleep apnea [3]. Bariatric surgery techniques produce significant improvements in serum lipids, but changes vary due to anatomic alterations distinct to each procedure. The RYGB leads to major improvements in blood glucose and insulin concentrations, hormonal responses, as well as decreased inflammatory markers [4,5]. Alterations in the secretion of gastric and intestinal peptides, such as glucagon-like peptide-1 (GLP-1), ghrelin and YY peptide were demonstrated after RYGB [6,7]. Lipid metabolism is also modified after surgery with decreased levels of total cholesterol and LDL-cholesterol, and increase in HDL-cholesterol [8]. Despite these effects in metabolic markers, the underlying mechanisms responsible for the observed changes remain to be defined.

The application of metabolomics and the possibility of profiling a large number of metabolites derived from different pathways provides new insights into metabolic interdependencies. As examples, metabolomics applications revealed the relationship between obesity and plasma branched-chain amino acids (BCAA) concentrations [9] and the association between plasma phospholipids with diabetes [10,11]. Although altered plasma lipid profiles have been related to obesity comorbidities such as diabetes, atherosclerosis and NAFLD, only a few studies have focused on the lipidome of patients undergoing RYGB [11,12,13,14]. 

In this study, changes in plasma lipid subclasses represented by acylcarnitines, phosphatidylcholines, sphingomyelins and bile acids were assessed before and after RYGB. We revealed important correlations between different lipid species and the major outcomes of the RYGB, with new insights into the metabolic changes after the surgery. Our approach involved the use of dried blood spots (DBS) to sample blood for metabolomics analysis. This practical sampling approach produced reliable data, similar to what have been previously described. Besides providing more insights into the mechanisms involved in RYGB effects on metabolism, this study also validates DBS as a sampling technique for clinical studies.

## 2. Material and Methods

### 2.1. Study Cohort and Sample Collection

Thirty-nine morbidly obese patients (14 male and 25 female subjects) as candidates for RYGB Surgery were recruited at Clinica IMEC (São Paulo, Brazil). All subjects gave written informed consent. Local ethics committee approved the clinical investigation (Protocol Number: 31498414.3.0000.0068). Exclusion criteria included patients younger than 18 or older than 60 years; patients with hepatopathies, pancreatopathies, inflammatory bowel disease, cancer, chronic diarrhea, unbalanced constipation (in regular use of laxatives), enterorrhagia; patients undergoing previous gastrointestinal surgeries. After the surgery, there was a drop out of 13 volunteers, leaving only 26 patients 15 and 90 days after surgery (7 males and 19 females). Table 1 presents the medication taken by some of the participants. Around 50% of the volunteers did not use any medication and only 2 patients took more than 2 different medications.

Dried blood spots (DBS) samples were collected after a 12-hours fasting 7 days before, 15 and 90 days after surgery by puncturing the tip of the patients’ finger with a sterile lancet on a blood collection filter paper for metabolomics analysis (903 Protein Saver, Whatman). Fasting plasma samples for clinical chemistry analysis were collected 7 days before and 90 days after surgery. Clinical chemistry and anthropometrics characteristics of the cohort are summarized in Table 2.

### 2.2. Biochemical Analyses

Serum glucose, HbA1c, triglycerides, total cholesterol, LDL and HDL-cholesterol, alanine aminotransferase (ALT), aspartate aminotransferase (AST), gamma-glutamyl transpeptidase (γGT), creatinine, uric acid, vitamin D, folic acid, ferritin, c-reactive protein, thyroxin and thyroid-stimulating hormone were measured in a certified clinical laboratory, using standard protocols.

### 2.3. Metabolite Profiling

In order to extract the metabolites from DBS, punches of 3 mm diameter (estimated total blood volume = 3.1 µL [15]) were soaked in 400 µL methanol for 30 minutes under agitation at room temperature. Methanolic extracts were filtered, evaporated and ressuspended in 100 µL methanol prior to analysis. The methanol used for the extraction contained a mix of internal standards for quantitation of the acylcarnitines (Chromsystems, Gräfelfing, Germany), phospholipids and sphingolipids (Avanti Polar Lipids, AL, USA) of interest.

Phosphatidylcholines, lysophosphatidylcholines and sphingomyelins were analyzed by mass spectrometry in FIA mode. For this, 10 µl of the extracts were injected into the triple quadrupole mass spectrometer (ABSciex 5500, Sciex USA) operated in positive mode [16]. Multiple reaction monitoring was performed using transmissions specific for each of the 76 phosphatidylcholines (PC), 18 lyso-phosphatidylcholines (L-PC) and 15 sphingomyelins (SM). The mobile phase consisted of LC-MS grade methanol:H2O (96.7:3.3) containing 5 mM NH_4_Ac, flowing at 35 uL/minute. Peak integration was performed with the Analyst 1.5 software (Sciex, Framingham, MA, USA) and metabolite concentrations calculated multiplying the ratio of metabolite: internal standard peak area by the concentration of the internal standards. Lyso PC a C9:0 and lyso PC a C19:0 were the internal standards used for Lyso-PC quantitation, while 3 different internal standards were used for PC quantitation in order to correct for chain length differences: PCaaC28:0, PCaaC40:0 and PCaa C46:0. Sphingomyelins were quantified using SM C6:0 as internal standard.

Acylcarnitines (AC) present in DBS methanolic extracts were quantified using LC-MS/MS, following the method of Giesbertz et al. [17]. In this analysis, 23 AC were quantified after butylation, using 13 deuterated acylcarnitines (or L-carnitine) as internal standards (d9-C0, d3-C2, d3-C3, d3-C4, d9-C5, d5-C5DC, d3-C6, d3-C8, d3-C10, d3-C12, d3-C14, d3-C16 and d3-C18).

The 10 most abundant bile acids (BA) present in the methanolic extracts of the DBS were quantified using an adaptation of the method described by Tagliacozzi et al. [18]. Bile acids were extracted from 3 punches of 3 mm diameter cut from the DBS, extracted as described above. In this case, the DBS methanolic extracts were mixed with a solution of deuterated internal standards, the solvent evaporated under N_2_ and the residue re-suspended in 100 µL methanol:water (1:1). LC-MS/MS analysis was performed using a triple quadrupole mass spectrometer (HPLC Agilent - CA, USA; QTrap 5500-ABSciex MA, USA) operated in negative mode and mass spectra acquired in multiple reaction monitoring mode. Bile acids were separated using a gradient with 0.2% formic acid in water and acetonitrile starting at 30% and increasing up to 100% at 10.5 minutes, with a flow rate of 0.6 mL/min. The RP column (Phenomenex Luna C18(2) 150 x 4,6 mm; 5 µm particle size) was kept at 40 °C. Analyst Software was used for peak integration. The solution of deuterated internal standards contained d4-Deoxycholic acid, d4-Glycoursodeoxycholic acid, d4-Glycodeoxycholic acid, d4-Glycocholic acid and d5-Taurocholic acid [19]. 

### 2.4. Statistical Analysis

Student’s T-test was used to assess differences in clinical parameters measured before and 90 days after the RYGB surgery. Mixed-effects analysis was employed for the comparisons of metabolite concentration between the 3 time points of sampling and Tukey’s test was used to correct for multiple comparisons. A correlation matrix between the metabolites with the highest VIP scores identified by the PLS-DA analysis and the clinical chemistry parameters was built using Spearman’s correlation. GraphPad Prism Software (version 8.0) was used for these analysis and preparing the figures. Multivariate data analysis was performed using Simca-P1 software (version 15.0.2; Umetrics, Umea, Sweden). The data was scaled using unit variance scaling. The separation of individuals based on metabolite profile (exclusively metabolomics data) differences induced by the RYGB surgery were explored using partial least-squares discriminant analysis (PLS-DA). The variable importance in projection (VIP) value of each variable was calculated to indicate their contribution to the classification of individuals. The quality of the model was judged by the goodness-of-fit parameter (R^2^), the predictive ability parameter (Q^2^), and the ANOVA analysis of the cross-validated residuals of the Y-variable. 

### 2.5. Ethical Approval

All procedures performed in studies involving human participants were in accordance with the ethical standards of the institutional (Protocol Number: 31498414.3.0000.0068) and with the 1964 Helsinki declaration and its later amendments or comparable ethical standards. Informed consent was obtained from all individual participants included in the study.

## 3. Results

### 3.1. Clinical Parameters

Anthropometric and clinical chemistry data are presented in Table 1. Prior to surgery, BMI was 44.1 ± 3.6 kg/m^2^ and 90 days after surgery it decreased to 35 ± 3.3kg/m2 (a decrease of 21%, *p* < 0.001). The RYGB’s favorable effect on serum lipid profile was also observed: cholesterol levels decreased by approximately 21% (*p* < 0.0001), LDL-C by 22% (*p* < 0.0001) and triacylglycerol by 29% (*p* = 0.0003). Glucose levels decreased by 17% and HbA1c level turned from 6.57 ± 2.2 before surgery to 5.5 ± 0.8 (*p* = 0.02).

### 3.2. Acylcarnitine Profile

**Figure 1:**
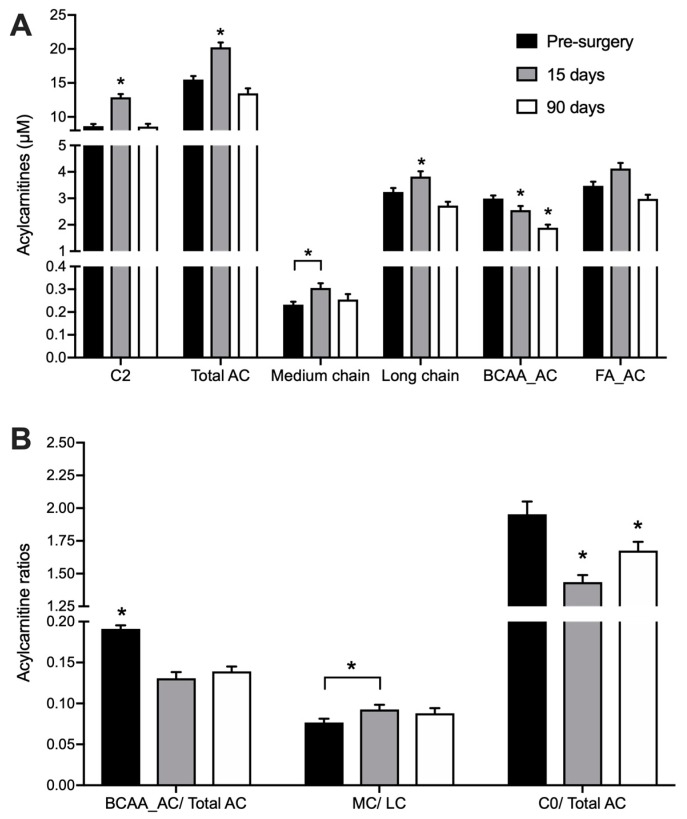
Acylcarnitines in DBS collected before, 15 and 90 days after RYGB. *n* = 26. * indicates *p* < 0.05 in comparison to all other groups, unless otherwise indicated. (**A**) C2: acetylcarnitine; Total AC: sum of all quantified acylcarnitines; medium chain: sum of acylcarnitines with 6 to 14 carbons; long chain: sum of acylcarnitines with 16–18 carbons; (**B**) BCAA_AC: sum of acylcarnitines derived from branched-chain amino acid catabolism (C3, 2M-C3, 2M-C4, 3M-C4 and C4DC); FA_AC = sum of medium-chain acylcarnitines derived from fatty acid catabolism (C6, C8, C10, C12, C14, C16, C16:1, C:18, C18:1, C18:2; C0 = L-carnitine.

We quantified 22 acylcarnitines (AC) species and L-carnitine in DBS samples (Table A1, Appendix). Total acylcarnitine concentration rose from 15.3 ± 0.6 µM at baseline to 19.1 ± 0.8 µM 15 days after surgery (*p* < 0.001) (Figure 1A). This effect was only transient as AC levels returned to initial values (14.3 ± 0.6 µM) 90 days after the procedure. The increase in AC levels observed 15 days after surgery were mainly due to acetylcarnitine (C2), hydroxybutyrylcarnitine (C4-OH) and long-chain AC species (mainly C16 and C18) - products of fatty acid (FA) catabolism. In contrast, RYGB surgery reduced the concentration of propionylcarnitine (C3) already 15 days after the procedure, and this change was still present after 90 days. Other acylcarnitines derived from branched-chain amino acids (BCAA) catabolism such as isovalerylcarnitine (3M-C4) and 2-methyl-butyrylcarnitine (2M-C4), had their concentrations decreased only 90 days after the surgery. The decrease in total BCAA-derived acylcarnitines (BCAA_AC) concentration 15 days after surgery was accentuated after 90 days with a 37% decrease (*p* < 0.05), leading to decreased ratios between these species and the sum of all acylcarnitines (Figure 1B).

Acylcarnitnies with even-carbon acyl chains ranging from C2 to C16 (as released into and measured in blood) are representative of their cognate acyl-CoA intermediates derived from FA β-oxidation. Therefore, the ratio between medium-chain (MC) and long-chain acylcarnitines (LC) may be used as an indicator of FA catabolism [20]. As expected, RYGB stimulates FA catabolism, leading to a progressive increase in the ratio of plasma MC/LC after surgery (Figure 1B). The ratio between free L-carnitine (C0) and the total pool of acylcarnitines could serve as an overall indicator of the catabolic state [21], since carnitine is conjugated to intermediates of FA and amino acid degradation. The ratio of C0/total AC decreased by 26% at 15 days after the surgery (*p* < 0.001) and was still lower than before surgery at day 90, indicating a sustained increased catabolism of endogenous substrates (Figure 1B). 

### 3.3. Bile Acids Profile

**Figure 2:**
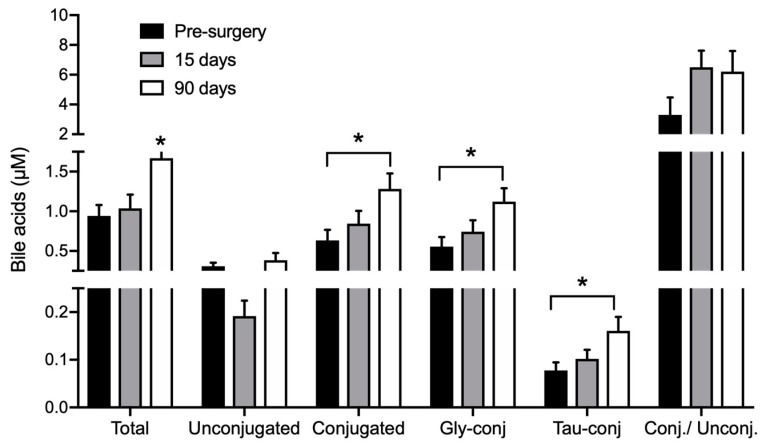
Bile acids in DBS collected before, 15 and 90 days after RYGB. *n* = 26. * indicates *p* < 0.05 compared to all groups, unless otherwise stated. Gly-conj: glycine-conjugated BA; Tau-conj: taurine-conjugated BA; Conj/Unconj: ratio between conjugated and unconjugated BA.

RYGB surgery led to increased BA concentrations in circulation, as summarized in Figure 2 and Table A2 (Appendix). At baseline, total BA concentration was 0.94 ± 0.13 µM and 90 days after surgery it was increased by 43% to 1.66 ± 0.21 µM (*p* < 0.05). This effect was mainly due to increased levels of glycine-conjugated BA species and led to an increase in the ratio between conjugated and unconjugated BA from 3.3 ± 1.2 before surgery to 6.2 ± 1.4 µM at day 90 (Figure 2). The concentration of glycochenodeoxycholic acid (GCDCA) increased 54% (*p* < 0.05) and glycocholic acid (GCA) acid by 43%. Taurochenodeoxycholic acid (TCDCA) also showed a significant increase of 57% (*p* < 0.05) (Table A2, Appendix). 

### 3.4. Phospholipid Profile

**Figure 3:**
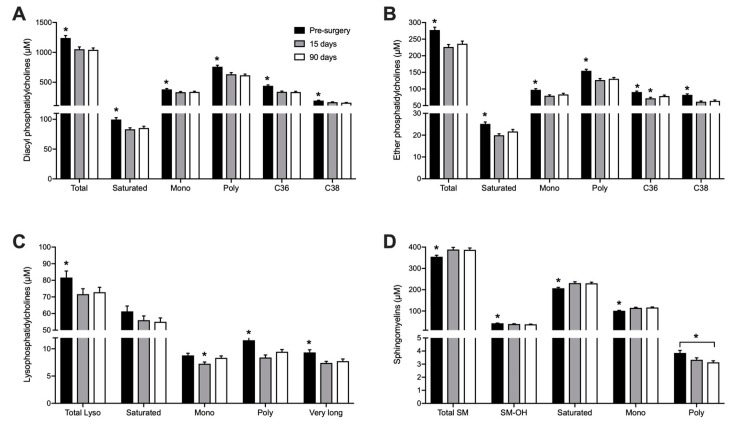
Whole blood concentration of Phosphatidylcholines (diacyl) (**A**), Ether-Phosphatidylcholines (**B**), Lysophosphatidylcholines (**C**) and Sphingomyelins (**D**) before, 15 and 90 days after RYGB. n = 26. * *p* < 0.05 when compared to all other groups, unless otherwise stated. Saturated: Sum of all molecules containing only saturated FA; mono: Sum of all molecules containing one monounsaturated FA; poly: Sum of all molecules containing at least one polyunsaturated FA; C36: sum of all PC species with 36 carbons; C38: Sum of all PC species with 38 carbons; very long: Lysophosphatidylcholines with 22–28 carbons; SM-OH: Sum of all sphingomyelins containing hydroxylated FA.

Seventy-six different phosphatidylcholines (PC), 18 lyso-phosphatidylcholines (L-PC) and 15 sphingomyelins (SM) were quantified (Table A3, Appendix). Phosphatidylcholines and sphingomyelins are the main phospholipids in mammalian plasma membranes and circulating lipoproteins. Whole blood samples collected in DBS are then expected to have much higher levels of these metabolites than plasma. Our method allowed the quantification of ester- and ether-linked phosphatidylcholines, and although ester-linked phospholipids are more abundant, both types presented a similar response pattern to RYGB surgery. Fifteen days after surgery, the concentration of total PC decreased by 15% (*p* < 0.001). As shown in Figure 3A,B, PCs containing both saturated and unsaturated FA showed lower concentration after surgery. PC-C36 and PC-C38, which contain long-chain FA (side chain of 16 to 20 carbons), represented more than 50% of total PCs quantified and were also the individual PC species with the most pronounced decrease 15 days after the surgery, with effects lasting up to 90 days.

Lysophosphatidylcholines (L-PC) result from hydrolysis of one acyl group of PC and represent intermediates in PC remodeling and synthesis. Similar to the PCs, L-PC concentrations in blood also diminished by 11% at day 15 (*p* < 0.01) and the effect was sustained 90 days after RYGB. The most pronounced decreased was noted in L-PC containing polyunsaturated and very long-chain FA, which decreased by 17% and 18% (*p* < 0.01), respectively (Figure 3C).

RYGB induced an opposite effect on sphingomyelins (SM) concentrations with a 10% increase 15 days after surgery (*p* < 0.001), with elevated levels remaining up to 90 days. This increase was observed in saturated and monounsaturated SM species, while SM containing polyunsaturated FA showed a decrease 90 days after the surgery (Figure 3D). 

### 3.5. Global Metabolic Responses to RYGB

**Figure 4:**
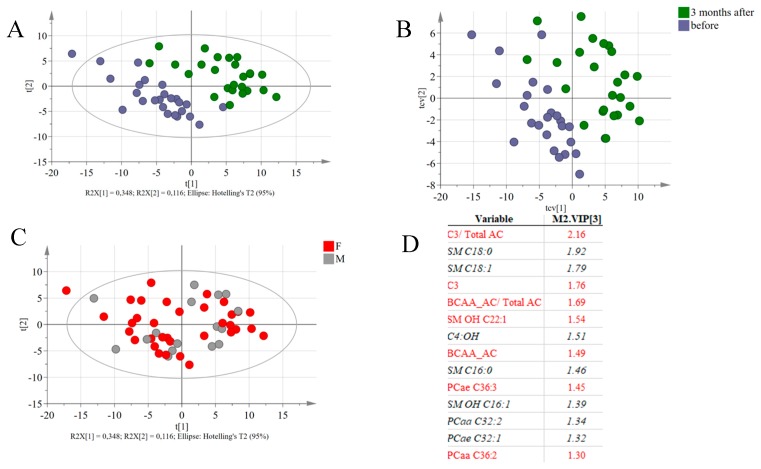
(**A**) Score plots of PLS-DA based on 143 identified lipid species from all patients before and 3 months after the surgery. (**B**) Cross-validation score plots, indicating that even after cross validation, the separation between the two groups of samples is still observed. (**C**) Score plots of the PLS-DA model, coloring the subjects according to gender. (**D**) List of variable with the highest VIP values. Variables in red are associated with samples collected before the intervention and variables in black and italic had higher concentrations in the samples collected 90 days after the intervention.

To assess major metabolic differences induced by the RYGB surgery, multivariate statistical analysis was performed based on DBS metabolite profiles (excluding anthropometric data, which are expected to change due to the procedure), resulting in a PLS-DA model with 3-components (R2 = 0.9 and Q2 = 0.74) (Figure 4A). Cross-validation of the model indicated its robustness (see cross-validation scores in Figure 4B) and the analysis of variance of the cross-validated residuals of the Y variable had a *p* value = 3,16.10–11. Both male and female participants were recruited to the study but the effect of gender was not evident in the model (Figure 4C). Metabolites with high VIP values are indicated as being responsible for the differences between samples collected before and 90 days after RYGB surgery (Figure 4D). Propionylcarnitine and other BCAA-derived AC, as well as the phosphatidylcholines PCae C36:3, PCaa C36:2 and sphingomyelin SM OH C22:1 had higher concentrations in samples collected before surgery, while SM 16:0, SM 18:0, SM 18:1, the acylcarnitine 3-Hydroxybutyrylcarnitine (C4:OH) and the phosphatidylcholines PCaa C32:2 and PCae C32:1 were increased in blood collected 90 days after the RYGB surgery. 

**Figure 5:**
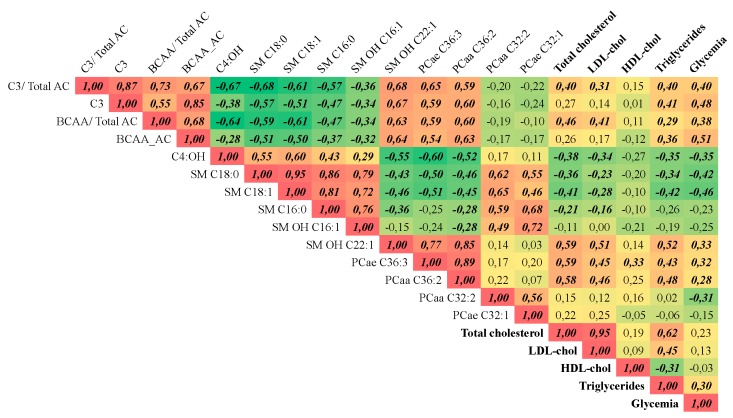
Correlation matrix. Spearman’s correlation of the variables with high VIP values in the PLS-DA model with clinical chemistry parameters. *n* = 26. Only data from before and three months after the RYGB surgery were used. r values in bold/ italic indicate that the correlation has a *p* < 0.05.C3 = propionyl carnitine; C3/ Total AC = ratio between C3 and the sum of all AC; BCAA_AC = AC derived from BCAA; BCAA_AC/total AC = ratio between the sum of AC derived from BCAA and the sum of all AC; C4:OH = 3-Hydroxybutyrylcarnitine; SM 18:0 = octadecanoyl sphingomyelin; SM 18:1 = oleoyl sphingomyelin; SM 16:0 = palmitoyl sphingomyelin;, SM OH 16:1 = Hydroxy-palmitoleyl sphingomyelin; SM OH 22:1 = Hydroxy-docosaenoicoyl sphingomyelin; PCae 36:3 = ether-linked phosphatidylcholine C36:3; PCaa 36:2 = phosphatidylcholine C36:2; PCaa 32:2 = phosphatidylcholine C32:2; PCae 32:1 = ether-linked phosphatidylcholine C32:2.

Spearman’s correlation analysis between metabolites with high VIP values and parameters of clinical chemistry indicated that these metabolites significantly correlated to alterations in cholesterol, triacylglycerol and glucose plasma concentrations (Figure 5). SM OH-C22:1 presented the highest positive correlation (r = 0.59) with total cholesterol, while the remaining SM species showed negative correlation. A similar relationship was observed between plasma triacylglycerol levels and these SM. LDL cholesterol levels also correlated positively with SM OH-C22:1 (r = 0.51). The ether-linked phosphatidylcholine C36:3 showed a significant positive correlation with total cholesterol (r = 0.59), LDL-cholesterol (r = 0.45,) and triacylglycerol (r = 0.43,). Glucose plasma concentration was positively correlated with propionylcarnitine (r = 0.48) and with the ratio of BCAA-derived acylcarnitines (r = 0.51), but negatively correlated to SM C18:0 and SM C18:1 (r = −0.42 and −0.46, respectively). Propionylcarnitine was also significantly correlated with triacylglycerol concentrations (r = 0.40). 

Apart from the correlations with clinical chemistry parameters, the above-mentioned sphingomyelins (except SM OH C22:1) were negatively correlated with BCAA-derived AC and PCs containing 36 carbons, but were positively correlated with the PCs containing 32 carbons (Figure 5).

## 4. Discussion

In the present study, we observed that RYGB has distinct effects on different lipid classes profiled in DBS. We are not aware of DBS being used before as a sampling technique in studies with patients undergoing bariatric surgery. Owing to their ease of collection, storage and shipment, DBS provide the option to assess plasma metabolite changes over time in an inexpensive and reliable manner. The metabolite changes detected by our method presented the same magnitude observed in previous studies [22,23,24]. In the present study, an increase in blood acylcarnitine levels at day 15 post surgery was observed, but levels fell again and returned almost to starting levels. On the other hand, an increase in bile acids concentration was observed three months after the surgery. A rapid and sustained decrease in phosphatidylcholines and an increase of sphingomyelins also occurred. 

Acylcarnitines are intermediaries of FA and amino acid catabolism. Carnitine availability is essential for these processes and depends on dietary intake and endogenous synthesis. Carnitine uptake and synthesis are regulated by PPAR-α and stimulated by dietary energy restriction [25]. In this study, the levels of C2 and other acylcarnitines, increased in blood 15 days after surgery, indicating increased use of endogenous substrates due to the lower caloric intake. However, their concentration returned to basal levels after 90 days, indicative of a physiological long-term adaptation. It has been previously shown that during dietary energy restriction, there is a decrease in energy expenditure [26,27] and Schooman et al observed a similar change in AC levels in subjects undergoing caloric restriction but not RYGB surgery [28].

Propionylcarnitine, isobutyrylcarnitine, 2-methyl-butyrylcarnitine, isovalerylcarnitine and methyl-malonylcarnitine are all products of BCAA catabolism and showed an accentuated decrease after 90 days, leading also to lower ratios between these metabolites and the sum of all acylcarnitines. In fact, reductions in BCAA one month after RYGB correlate with improvement in glucose homeostasis ^11^. Although we have not quantified amino acids, other studies reported decrease in their concentrations after RYGB [29,30]. The concentration of BCAA-derived AC has been positively correlated with obesity and insulin resistance in different studies and that seems to originate from an altered BCAA catabolism in adipose tissue. It seems that with their increase in circulation, BCAA become available for oxidation in liver and skeletal muscle, leading to increased concentration of the acylcarnitines derived from BCAA catabolism in obese patients [9,31]. In the present as well in previous studies, there is a positive correlation between propionylcarnitine and glucose levels (r = 0.3) as shown in Figure 4B, suggesting an interplay in utilization of BCAA and glucose. Altered changes in BCAA-derived AC after bariatric surgery suggest that the overall oxidation of BCAA is increased and associated with reduced levels of BCAA in circulation.

Bile acid metabolism is crucial to cholesterol homeostasis and BA modulate metabolic processes through G protein-coupled receptor (TGR5) and nuclear receptors (FXR, LXR and others) [32]. Previous reports describe increased plasma BA concentrations in patients undergoing RYGB surgery [33,34] related to improvements in glucose and lipid metabolism. The activation of hepatic nuclear receptors by BA stimulates lipid oxidation and may protect obese patients from hepatic steatosis and hepatic insulin resistance [35]. The activation of TGR5 receptors in colonic endocrine cells can stimulate GLP-1 secretion, improving glucose and lipid homeostasis [32]. The stimulation of FGF19 secretion by BA may directly affect liver lipid oxidation [36]. De Giorgi et al also showed that faster absorption of dietary triglycerides after RYGB surgery may be responsible for decreased postprandial and basal triglyceride concentrations observed in these patients [37]. The proposed mechanisms mediating such effects involve a pronounced early peak of FGF19 and apoB48 availability associated with the BA changes, leading to faster clearance of triglycerides. A reduced postprandial increase of conjugated but not unconjugated BA in the plasma of morbidly obese patients has been reported. Therefore, a RYGB-induced normalization of BA profiles may contribute to the effects of RYGB in energy expenditure [38]. In our study, the higher increases detected in conjugated BA species may be related to alterations in gut microbiota following RYGB, as previously reported [39]. These changes are consistent with the data of Tremaroli et al. [40] observing an increase in microbial gene expression of enzymes involved in BA dehydroxylation after RYGB. Indeed, a recent study described microbial-related metabolites such as secondary BA and hydroxylated fatty acids as biomarkers of a stable metabolic state following bariatric surgery, independent of the patients health status at baseline [41].

Phospholipids and sphingolipids correspond to a large portion of the plasma lipidome. Plasma phospholipid concentrations are modulated by dietary and adipose tissue changes [42]. In our study, we observed a marked decrease in the concentration of PC containing long-chain and very long-chain FAs. Forbes et al. [43] have also shown that the content of linoleic acid (C18:2) within plasma phospholipids was decreased in patients 1 and 6 months after RYGB, presumably due to reduction of dietary fat intake [44,45] or due to increased FA ß-oxidation [45]. Graessler et al found significant decrease in several phospholipids classes in plasma, including ceramides, lysoPCs, PCs and ether PCs 3 months after RYGB, without changes in SM levels [12]. Luo P et al. [24] found decreased PC, LysoPC species and non-esterified FA 6 and 12 months after RYGB. They also found changes in some SM species, such as an increase in SM16:0 and a decrease in SM20:0 concentration. Mutch et al. [46] observed increases in some sphingosines, unsaturated FAs, and phospholipids, while ceramide, triglycerides, and saturated FAs decreased following RYGB. What makes our study unique is that the phospholipids analyzed comprise not only of those found in plasma but also those in blood cell membranes and in lipoproteins; nevertheless, the reported changes go in the same direction as most of the previously published studies. 

Sphingomyelins amongst all metabolites analyzed correlated best with clinical parameters. Whilst the majority of SM with high VIP values displayed negative correlations, SM OH C22:1 displayed a positive correlation with total cholesterol (r = 0.59), LDL cholesterol (r = 0.51) and triglycerides (r = 0.52) levels. SM are known to be constituents of membranes of HDL particles and their accumulation decreases the fluidity of the lipid monolayer, affecting the metabolism of lipoproteins [47,48]. Dietary sphingomyelin (SM) was shown to reduce the absorption of cholesterol, triglycerides and FAs in vitro [49] and in rat models [50]. Thus, changes in SM species may be secondarily related to changes in cholesterol and triglycerides homeostasis or may even be primarily involved in the metabolic adaptation following bariatric surgery. 

The reported changes in blood lipid profiles described here call for more research on the chronology of metabolic adaptations to RYGB surgery to better understand how the rapid improvements in health status of patients are achieved. Follow-up studies should control for energy and nutrient intake, although it is impossible to completely separate the diet restriction effect intrinsic to this kind of surgery, to sort out whether exogenous (diet) or endogenous factors are the main causes of the changes in blood lipid entities described here.

## Figures and Tables

**Table 1:** Medications taken by the patients and medical conditions reported at baseline.

Medications	Number of Patients
Statins	2
Antihypertensives	7
Insulin	1
Metformin	3
Levothyroxine	4
**Conditions**	**-**
Tabagism	7
Low-grade/moderate steatosis	17

Only two patients took more than 2 medications. Around 50% of the volunteers did not use any medication.

**Table 2:** Clinical data before and 90 days after RYGB surgery. * *p* < 0.05, ** *p* < 0.01, *** *p* < 0.001.

	Pre-Surgery *n* = 26	90 Days after Surgery *n* = 26
Age	36.2 ± 7.8	-
Sex (Fem/Male)	18/8	-
Weight (kg)	122 ± 22.4	96 ± 16.4 ***
% Weight loss		20.9 ± 6.1 ***
BMI (kg/m^2^)	44.1 ± 3.6	35 ± 3.3 ***
% BMI loss	-	20.7 ± 6.2 ***
Waist circ. (cm)	121.5 ± 17.4	104.8 ± 12.6 **
Hip circ. (cm)	133.1 ± 11.6	122.3 ± 15.9 **
Glucose (mg/dL)	101.2 ± 22.5	84.36 ± 8.6 *
HbA1c (%)	6.57 ± 2.2	5.5 ± 0.8 *
Cholesterol (mg/dL)	203.68 ± 45.4	161.8 ± 40.3 ***
LDL-C (mg/dL)	126 ± 38.9	98.7 ± 32.6 ***
HDL-C (mg/dL)	42.7 ± 9.6	38.52 ± 10.2 *
Triglycerides (mg/dL)	169.9 ± 97.2	121.3 ± 87.8 ***
γGT (U/L)	41.8 ± 22.4	25.6 ± 19.4 *
ALT (U/L)	23.2 ± 10.9	20.7 ± 6.3
AST (U/L)	35.2 ± 25.6	38.6 ± 42.6
Creatinine (mg/dL)	0.81 ± 0.15	0.71 ± 0,1 *
Uric acid (mg/dL)	6.1 ± 2.1	5.1 ± 1.4 **
Vit D (pg/dL)	23.2 ± 7.8	24.6 ± 7.8
Folic acid (ng/dL)	10.4 ± 3	10.2 ± 5.4
Ferritin (ng/dL)	156 ± 130.5	150.2 ± 101.3
PCR (mg/dL)	5.01 ± 5.9	4.65 ± 8.5
T4 (ug/dL)	8.3 ± 1.8	7.5 ± 2.5
TSH (mU/L)	3.8 ± 4.6	2.8 ± 2.5

## Abbreviations

RYGB: Roux-en-Y gastric bypass; GLP-1: glucagon-like peptide-; BCAA: branched-chain amino acids; AC: acylcarnitines; PC: phosphatidylcholines; L-PC: Lysophosphatidylcholines; SM: sphingomyelins; BA: bile acids; DBS: dried blood spots; FA: fatty acid; BCAA_AC: BCAA-derived acylcarnitines; MC_AC: Medium-chain acylcarnitines; LC_AC: long-chain acylcarnitines, C0: L-carnitine.

## Appendix A

**Table A1:** Acylcarnitine concentrations (µM) in dried blood spots form patients before, 15 days and 90 days after RYGB surgery. total: sum of all quantified acylcarnitines; Medium chain (MC): sum of acylcarnitines with 6 to 14 carbons; Long chain (LC): sum of acylcarnitines with 16–18 carbons; BCAA-derived: sum of acylcarnitines derived from branched-chain amino acid catabolism (C3, 2M-C3, 2M-C4, 3M-C4 and C4DC); FA-derived = sum of medium-chain acylcarnitines derived from fatty acid catabolism (C6, C8, C10, C12, C14, C16, C16:1, C:18, C18:1, C18:2; C0 = L-carnitine. Values represent mean ± SEM. ^a^ *p* < 0.05 compared to pre-surgery; ^b^ *p* < 0.05 compared to 15 days.

Metabolites	Pre-Surgery	15 Days After	90 Days After
L-carnitine	30.034 ± 1.764	27.302 ± 1.583	23.611 ± 1.117 ^a,b^
C4-OH	0.027 ± 0.001	0.217 ± 0.022 ^a^	0.06 ± 0.004 ^a,b^
C2	8.756 ± 0.369	11.956 ± 0.526 ^a^	8.81 ± 0.485 ^b^
C5-OH	0.222 ± 0.023	0.232 ± 0.021	0.164 ± 0.019 ^a,b^
C3	1.617 ± 0.084	1.224 ± 0.119 ^a^	0.855 ± 0.051 ^a,b^
2M-C3	0.116 ± 0.009	0.183 ± 0.015 ^a^	0.098 ± 0.006 ^b^
C4	0.058 ± 0.008	0.05 ± 0.006 ^a^	0.041 ± 0.004 ^a,b^
2M-C4	0.064 ± 0.004	0.07 ± 0.005	0.039 ± 0.002 ^a,b^
3M-C4	0.112 ± 0.006	0.119 ± 0.009	0.089 ± 0.006 ^a,b^
C4DC	1.038 ± 0.069	0.881 ± 0.072 ^a^	0.882 ± 0.072 ^a^
C3DC-M	0.012 ± 0.001	0.009 ± 0.001 ^a^	0.011 ± 0
C6	0.034 ± 0.003	0.041 ± 0.003	0.034 ± 0.002
C5DC	0.029 ± 0.003	0.021 ± 0.002 ^a^	0.028 ± 0.002 ^b^
C6DC	0.007 ± 0.001	0.027 ± 0.003 ^a^	0.014 ± 0.001 ^a,b^
C8	0.032 ± 0.003	0.045 ± 0.004 ^a^	0.042 ± 0.004
C10	0.068 ± 0.007	0.1 ± 0.011 ^a^	0.097 ± 0.013 ^a^
C12	0.033 ± 0.002	0.042 ± 0.004 ^a^	0.036 ± 0.002
C14	0.068 ± 0.004	0.069 ± 0.005	0.054 ± 0.002 ^a,b^
C16:1	0.092 ± 0.005	0.127 ± 0.012 ^a^	0.098 ± 0.005 ^b^
C18:2	0.445 ± 0.031	0.456 ± 0.03	0.374 ± 0.026 ^a,b^
C16	1.177 ± 0.075	1.295 ± 0.095 ^a^	1.039 ± 0.044 ^a,b^
C18:1	1.002 ± 0.063	1.33 ± 0.091 ^a^	1.043 ± 0.055 ^b^
C18	0.438 ± 0.037	0.37 ± 0.035 ^a^	0.329 ± 0.023 ^a^

**Table A2:** Bile acid concentration (µM) in dried blood spots from patients before, 15 days and 90 days after RYGB. CDCA: chenodeoxycholic acid; DCA: deoxycholic acid; CA: cholic acid; GCDCA: glycochenodeoxycholic acid; GDCA: glycodeoxycholic acid; GUDCA: glycoursodeoxycholic acid; GCA: glycocholic acid; TCDCA: taurochenodeoxycholic acid; TDCA: taurodeoxycholic acid; TUDCA: tauroursodeoxycholic acid; TCA: taurocholic acid. Primary: sum of CDCA, CA, GCDCA, GCA, TCDCA and TCA; Secondary + Tertiary: sum of DCA, GDCA, GUDCA, TDCA and TUDCA; Glyco: sum of all glycine-conjugated BA; Tauro: Sum of all taurine-conjugated BA. Values represent mean ± SEM. ^a^ *p* < 0.05 compared to pre-surgery; ^b^ *p* < 0.05 compared to 15 days.

Metabolites	Pre-Surgery	15 Days After	90 Days After
CDCA	0.117 ± 0.023	0.099 ± 0.023	0.118 ± 0.034
DCA	0.155 ± 0.02	0.089 ± 0.015 ^a^	0.183 ± 0.024 ^b^
CA	0.045 ± 0.013	0.035 ± 0.007	0.092 ± 0.047
GCDCA	0.315 ± 0.068	0.441 ± 0.088	0.688 ± 0.102 ^a^
GDCA	0.097 ± 0.02	0.135 ± 0.033	0.205 ± 0.053
GUDCA	0.033 ± 0.006	0.058 ± 0.015	0.034 ± 0.008
GCA	0.109 ± 0.032	0.107 ± 0.024	0.194 ± 0.03
TCDCA	0.039 ± 0.009	0.055 ± 0.011	0.091 ± 0.018 ^a^
TDCA	0.014 ± 0.003	0.02 ± 0.005	0.034 ± 0.01
TUDCA	0.002 ± 0.0007	0.002 ± 0.0008	0.001 ± 0.0001
TCA	0.022 ± 0.005	0.024 ± 0.004	0.033 ± 0.004 ^a^

**Table A3:** Glycerophospholipids and sphingomyelins concentration (µM) in dried blood spots collected from patients before, 15 days and 90 days after RYGB surgery. ^a^ *p* < 0.05 compared to pre-surgery. ^b^ *p* < 0.05 compared to 15 days.

Metabolites	Pre-Surgery	15 Days After	90 Days After
*Lysophosphatidylcholines*			
Lyso PC a C14:0	2.16 ± 0.07	1.7 ± 0.04 ^a^	1.78 ± 0.07 ^a^
Lyso PC a C16:0	39.61 ± 2.21	40.09 ± 2.11	37.69 ± 1.67
Lyso PC a C16:1	1.07 ± 0.07	0.68 ± 0.04 ^a^	0.85 ± 0.05 ^a,b^
Lyso PC a C17:0	1.15 ± 0.04	1.09 ± 0.04	1.11 ± 0.05
Lyso PC a C18:0	12.67 ± 0.72	8.47 ± 0.48 ^a^	9.72 ± 0.46 ^a,b^
Lyso PC a C18:1	5.73 ± 0.27	4.7 ± 0.26 ^a^	5.69 ± 0.25 ^b^
Lyso PC a C18:2	7.94 ± 0.41	4.96 ± 0.35 ^a^	6.24 ± 0.28 ^a,b^
Lyso PC a C18:3	0.25 ± 0.01	0.12 ± 0.01 ^a^	0.15 ± 0.01 ^a,b^
Lyso PC a C20:3	0.79 ± 0.05	0.39 ± 0.02 ^a^	0.47 ± 0.03 ^a,b^
Lyso PC a C20:4	1.72 ± 0.09	1.93 ± 0.11	1.8 ± 0.1
Lyso PC a C20:5	0.54 ± 0.02	0.46 ± 0.02 ^a^	0.42 ± 0.02 ^a^
Lyso PC a C22:5	0.17 ± 0.01	0.14 ± 0.01	0.16 ± 0.01
Lyso PC a C22:6	0.36 ± 0.02	0.38 ± 0.02	0.34 ± 0.02 ^b^
Lyso PC a C24:0	1.61 ± 0.07	1.48 ± 0.06	1.4 ± 0.06 ^a^
Lyso PC a C26:0	2.99 ± 0.18	2.13 ± 0.1 ^a^	2.26 ± 0.15 ^a^
Lyso PC a C26:1	0.92 ± 0.03	0.83 ± 0.03	0.85 ± 0.03
Lyso PC a C28:0	2.23 ± 0.14	1.43 ± 0.07 ^a^	1.61 ± 0.1 ^a^
Lyso PC a C28:1	1.24 ± 0.07	1.07 ± 0.05	1.06 ± 0.06 ^a^
Total Lyso PC	83.14 ± 3.8	72.05 ± 3.38 ^a^	73.52 ± 2.99 ^a^
*Diacyl Phosphatidylcholines*			
PC aa C24:0	1.63 ± 0.08	1.6 ± 0.06	1.44 ± 0.05 ^a^
PC aa C26:0	3.25 ± 0.14	2.66 ± 0.11 ^a^	2.85 ± 0.18
PC aa C28:1	3.13 ± 0.1	2.63 ± 0.1 ^a^	2.62 ± 0.09 ^a^
PC aa C30:0	5.96 ± 0.21	4.93 ± 0.17 ^a^	5.17 ± 0.15 ^a^
PC aa C30:2	5.24 ± 0.15	5.7 ± 0.22	5.51 ± 0.18
PC aa C32:0	33 ± 0.92	33.68 ± 1.08	34.68 ± 0.78
PC aa C32:1	23.49 ± 1.22	20.82 ± 0.79	22.49 ± 0.92
PC aa C32:2	6.6 ± 0.2	7.1 ± 0.31	7.1 ± 0.22
PC aa C32:3	0.79 ± 0.03	0.82 ± 0.04	0.79 ± 0.03
PC aa C34:1	215.38 ± 9.04	201.78 ± 7.9	204.45 ± 7.12
PC aa C34:2	260.09 ± 9.01	218.43 ± 11.39 ^a^	218.85 ± 9.12 ^a^
PC aa C34:3	9.48 ± 0.36	5.1 ± 0.24 ^a^	6.37 ± 0.38 ^a,b^
PC aa C34:4	0.95 ± 0.05	0.46 ± 0.02 ^a^	0.54 ± 0.03 ^a,b^
PC aa C36:0	36.82 ± 1.39	28.37 ± 1.25 ^a^	30.02 ± 1.49 ^a^
PC aa C36:1	91.27 ± 3.25	65.32 ± 2.33 ^a^	68.49 ± 3.09 ^a^
PC aa C36:2	164.82 ± 6.44	96.06 ± 4.7 ^a^	111.44 ± 5.75 ^a,b^
PC aa C36:3	72.14 ± 3.49	46.63 ± 2.45 ^a^	50.28 ± 2.48 ^a^
PC aa C36:4	73.74 ± 4.01	98.21 ± 6.19 ^a^	69.46 ± 2.28 ^b^
PC aa C36:5	5.67 ± 0.43	3.5 ± 0.2 ^a^	3.55 ± 0.18 ^a^
PC aa C36:6	1.32 ± 0.05	1.06 ± 0.04 ^a^	1.17 ± 0.06 ^b^
PC aa C38:0	18.12 ± 0.76	10.17 ± 0.46 ^a^	11.91 ± 0.73 ^a,b^
PC aa C38:1	45.05 ± 1.16	36.62 ± 1.14 ^a^	34.54 ± 1.34 ^a^
PC aa C38:3	43.04 ± 1.72	30.8 ± 1.22 ^a^	32.09 ± 1 ^a^
PC aa C38:4	50.57 ± 2.36	47.66 ± 2.67	42.51 ± 1.57 ^a^
PC aa C38:5	19.43 ± 0.88	18.12 ± 0.98	17.73 ± 0.63
PC aa C38:6	19.29 ± 0.74	22.87 ± 1.45 ^a^	18.51 ± 0.61 ^b^
PC aa C40:1	6.12 ± 0.2	5.23 ± 0.16 ^a^	4.98 ± 0.15 ^a^
PC aa C40:2	4.23 ± 0.17	3.82 ± 0.14 ^a^	3.85 ± 0.17 ^a^
PC aa C40:3	3.82 ± 0.12	3.24 ± 0.1 ^a^	3.39 ± 0.09 ^a^
PC aa C40:4	3.86 ± 0.15	3.14 ± 0.11 ^a^	3.03 ± 0.09 ^a^
PC aa C40:5	5.73 ± 0.26	4.04 ± 0.19 ^a^	4.37 ± 0.18 ^a^
PC aa C40:6	9.69 ± 0.39	7.48 ± 0.38 ^a^	7.3 ± 0.3 ^a^
PC aa C42:0	2.4 ± 0.09	2.04 ± 0.07 ^a^	2.03 ± 0.08 ^a^
PC aa C42:1	1.91 ± 0.07	1.65 ± 0.06 ^a^	1.71 ± 0.07 ^a^
PC aa C42:2	1.44 ± 0.06	1.41 ± 0.06	1.33 ± 0.05 ^a^
PC aa C42:4	0.74 ± 0.03	0.61 ± 0.02 ^a^	0.62 ± 0.02 ^a^
PC aa C42:5	0.91 ± 0.04	0.85 ± 0.03	0.8 ± 0.03 ^a^
PC aa C42:6	2.24 ± 0.09	2.32 ± 0.09	2.05 ± 0.1 ^b^
Total PC	1253.36 ± 40.09	1046.94 ± 39.25 ^a^	1036.32 ± 31.58 ^a^
*Ether Phosphatidylcholines*			
PC ae C30:0	1.18 ± 0.04	0.88 ± 0.03 ^a^	0.96 ± 0.04 ^a^
PC ae C30:1	2.62 ± 0.07	2.54 ± 0.09	2.53 ± 0.08
PC ae C30:2	1.04 ± 0.05	0.91 ± 0.04	0.87 ± 0.03 ^a^
PC ae C32:1	3.95 ± 0.11	4.11 ± 0.15	4.38 ± 0.14 ^a^
PC ae C32:2	1.02 ± 0.04	0.96 ± 0.03	1 ± 0.04
PC ae C34:0	5.43 ± 0.16	4.79 ± 0.16 ^a^	4.76 ± 0.14 ^a^
PC ae C34:1	11 ± 0.37	9.61 ± 0.4 ^a^	10.55 ± 0.38 ^b^
PC ae C34:2	7.81 ± 0.2	5.34 ± 0.22 ^a^	6.71 ± 0.23 ^a,b^
PC ae C34:3	3.46 ± 0.11	2.44 ± 0.11 ^a^	3.36 ± 0.11 ^b^
PC ae C36:0	9.12 ± 0.37	7.76 ± 0.32 ^a^	8.71 ± 0.43 ^b^
PC ae C36:1	38.55 ± 1.41	30.91 ± 1.33 ^a^	33.67 ± 1.53 ^a^
PC ae C36:2	28.54 ± 1.05	21.57 ± 0.96 ^a^	23.54 ± 1.18 ^a^
PC ae C36:3	5.15 ± 0.14	3.27 ± 0.14 ^a^	3.8 ± 0.12 ^a,b^
PC ae C36:4	7.04 ± 0.27	5.43 ± 0.28 ^a^	5.65 ± 0.17 ^a^
PC ae C36:5	3.65 ± 0.18	2.93 ± 0.16 ^a^	3.54 ± 0.15 ^b^
PC ae C38:0	6.75 ± 0.26	4.48 ± 0.18 ^a^	5.16 ± 0.29 ^a,b^
PC ae C38:1	21.9 ± 0.86	13.51 ± 0.58 ^a^	15.04 ± 0.87 ^a^
PC ae C38:2	20.78 ± 0.81	12.98 ± 0.52 ^a^	14.92 ± 0.83 ^a,b^
PC ae C38:3	12.32 ± 0.46	11.25 ± 0.53	10.29 ± 0.37 ^a^
PC ae C38:4	8.86 ± 0.32	8.23 ± 0.42	7.2 ± 0.18 ^a,b^
PC ae C38:5	6.3 ± 0.24	5.99 ± 0.34	5.91 ± 0.16
PC ae C38:6	6.52 ± 0.2	5.15 ± 0.18 ^a^	5.6 ± 0.22 ^a^
PC ae C40:1	14.42 ± 0.59	13.98 ± 0.56	12.48 ± 0.42 ^a,b^
PC ae C40:2	10.94 ± 0.38	11.01 ± 0.41	10.01 ± 0.31
PC ae C40:3	6.88 ± 0.27	5.97 ± 0.24 ^a^	5.73 ± 0.19 ^a^
PC ae C40:4	4.41 ± 0.17	3.72 ± 0.17 ^a^	3.57 ± 0.12 ^a^
PC ae C40:5	3.88 ± 0.13	3.94 ± 0.22	3.66 ± 0.12
PC ae C40:6	2.93 ± 0.08	2.19 ± 0.07 ^a^	2.21 ± 0.14 ^a^
PC ae C42:0	3.11 ± 0.15	2.11 ± 0.08 ^a^	2.13 ± 0.11 ^a^
PC ae C42:1	6.57 ± 0.31	4.94 ± 0.19 ^a^	5 ± 0.22 ^a^
PC ae C42:2	4.73 ± 0.2	4.03 ± 0.15 ^a^	4.07 ± 0.16 ^a^
PC ae C42:3	2.81 ± 0.11	2.6 ± 0.1	2.54 ± 0.09 ^a^
PC ae C42:4	1.61 ± 0.05	1.36 ± 0.06 ^a^	1.32 ± 0.05 ^a^
PC ae C42:5	1.77 ± 0.05	1.52 ± 0.06 ^a^	1.49 ± 0.05 ^a^
PC ae C44:3	0.86 ± 0.04	0.69 ± 0.03 ^a^	0.72 ± 0.03 ^a^
PC ae C44:4	0.72 ± 0.03	0.62 ± 0.02 ^a^	0.62 ± 0.02 ^a^
PC ae C44:5	1.01 ± 0.03	0.97 ± 0.04	0.86 ± 0.03 ^a,b^
PC ae C44:6	1.33 ± 0.05	1.17 ± 0.04 ^a^	1.07 ± 0.04 ^a^
Total ether PC	280.94 ± 8.18	71.97 ± 2.56 ^a^	68.98 ± 2.17 ^a^
*Sphingomyelins*			
SM OH C14:1	6.93 ± 0.22	7.18 ± 0.26	7.03 ± 0.26
SM OH C16:1	4.77 ± 0.14	5.9 ± 0.22 ^a^	5.86 ± 0.21 ^a^
SM OH C22:1	15.81 ± 0.48	11.65 ± 0.38 ^a^	10.6 ± 0.43 ^a,b^
SM OH C22:2	9.61 ± 0.23	9 ± 0.29	8.38 ± 0.24 ^a^
SM OH C24:1	5.81 ± 0.16	5.67 ± 0.19	5.27 ± 0.15 ^a^
SM C16:0	120.73 ± 2.77	136.64 ± 3.85 ^a^	141.02 ± 3.18 ^a^
SM C16:1	14.83 ± 0.39	17.92 ± 0.59 ^a^	16.56 ± 0.48 ^a^
SM C18:0	24.59 ± 0.73	34.76 ± 1.21 ^a^	35.81 ± 1.21 ^a^
SM C18:1	11.5 ± 0.39	18.74 ± 0.8 ^a^	17.43 ± 0.66 ^a^
SM C20:2	0.83 ± 0.03	0.78 ± 0.03	0.81 ± 0.02
SM C22:3	3.05 ± 0.18	2.53 ± 0.13 ^a^	2.27 ± 0.09 ^a^
SM C24:0	58.58 ± 1.66	55.81 ± 1.93	48.37 ± 1.48 ^a,b^
SM C24:1	71.86 ± 1.66	74.49 ± 2.19	77.58 ± 1.82 ^a^
SM C26:0	3.88 ± 0.09	3.66 ± 0.12	3.56 ± 0.09 ^a^
SM C26:1	3.57 ± 0.09	3.49 ± 0.11	3.75 ± 0.09 ^b^
Total SM	356.34 ± 6.91	462.32 ± 12.25 ^a^	453.78 ± 8.33 ^a^
